# 

**Published:** 1896-10

**Authors:** 


CONTENTS—OCTOBER, 1896.—VOL. XII., NO. 4.

ORIGINAL CONTRIBUTIONS—
Chronic Interstitial Nephritis. J. A. Gillis, M. D. . . . . . . . . . . 161
Chronic Constipation. J. W. Kinney, M. D . . . . . . . . . . . . 166
Osteo-myelitis of the Tibia and Fibula. E. B. Osborn, M. D . . . . 169
The Cure of Deviations of the Nasal Septum. Vard H. Hulen, M. D. 173
Modern Treatment of Acute Inflammation of the Middle Ear. J. A. Mullen, M. D . . . . . . . . . . . . . . . . . . . . . . . . . 175
CORRESPONDENCE—
Serum Therapy Antedated. Letter from Dr. J. W. Daniel. Recent Meeting of the American Ophthalmological Society . . . . . . 178–181
SOCIETY NOTES—
Brazos Valley Medical Association. Tri-State Medical Society of Alabama, Georgia and Tennessee. Transportation Arrangements for the Meeting of the Pan-American Medical Congress . . . . . . 183–186
ABSTRACTS AND SELECTIONS—
Sero-Therapy in Tuberculosis . . . . . . . . . . . . . . . . . . 186
Some of the Unsuspected Effects of the Bromides. J. Allison Hodges, M. D. . . . . . . . . . . . . . . . . . . . . . . . . 189
EDITORIAL—
The Medical Law.—A Suggestion for Relief . . . . . . . . . . . . 197
Where is the Loop-hole . . . . . . . . . . . . . . . . . . . 200
Some More Quackery and Bogus Diplomas . . . . . . . . . . . 203
MEDICAL NEWS AND MISCELLANY . . . . . . . . . . . . . . 204–211
Journal's Own Page . . . . . . . . . . . . . . . . . . . . . . 211
THE JOURNAL'S EXCHANGE BUREAU . . . . . . . . . . . . . . 211
BOOK NOTICES . . . . . . . . . . . . . . . . . . . . . . . 212–217
PUBLISHERS' NOTES . . . . . . . . . . . . . . . . . . . . 217–224

